# Sports Participation and Anti-Epidemic: Empirical Evidence on the Influence of Regular Physical Activity on the COVID-19 Pandemic in Mainland China

**DOI:** 10.3390/ijerph191710689

**Published:** 2022-08-27

**Authors:** Ruofei Lin, Xiaoli Hu, Shijun Chen, Junpei Huang

**Affiliations:** 1 School of Economics and Management, Tongji University, Shanghai 200092, China; 2 International College of Football, Tongji University, Shanghai 200092, China

**Keywords:** physical activity, COVID-19, immunity, ordinary least squares regression, instrumental variable, mediating effect model

## Abstract

This study aims to investigate the effects and influencing mechanisms of regular physical activity (RPA) on the COVID-19 pandemic. Daily data from 279 prefecture-level cities in mainland China were collected from 1 January to 17 March 2020. A two-way fixed-effects model was used to identify the causal relationship between physical activity and COVID-19, while also considering factors such as patterns of human behavior and socioeconomic conditions. The instrumental variable (IV) approach was applied to address potential endogeneity issues for a more accurate causal identification, and the mediating effect model was applied to examine the mechanisms of the influence of physical activity on the epidemic. We found that regular physical activity significantly improves individual immunity, which, in turn, leads to a reduction in the probability of being infected with COVID-19. Furthermore, we investigated the heterogeneity of the influence, finding that the negative impact of physical activity on the pandemic is more pronounced in the absence of adequate medical resources, strong awareness of prevention among residents, and fully implemented public health measures. Our results provide empirical evidence for the mechanisms of influence of physical activity on the pandemic. We would suggest that not only should physical activity be actively practiced during the pandemic, but also long-term regular exercise habits should be consciously cultivated to improve the ability of the individual immune system to better cope with sudden outbreaks of emerging infectious diseases.

## 1. Introduction

As a global public health crisis, since the outbreak of the COVID-19 pandemic (caused by the virus known as SARS-CoV-2), COVID-19 has rapidly spread and infected more than 460 million people worldwide. Although the majority of people infected with the virus are asymptomatic or have mild symptoms [1], there are still many serious sequelae [2] that pose a serious threat to human health. In addition, numerous widespread variants of COVID-19 have been identified worldwide, including Delta (B.1.617.2) [3] and Omicron (B.1.1.529) [4]. It should be recognized that COVID-19 is likely to coexist with humans in the long term, and that other unpredictable variants may emerge, leading to a new pandemic. Therefore, it is necessary to explore the long-term mechanisms for effective epidemic prevention and control. In addition to timely pharmacological interventions and public health measures, improving individual immunity plays an important role. Particularly when new variants emerge, it may not be possible to ensure rapid access to effective pharmacological interventions such as vaccines, and it is difficult for governments to quickly implement comprehensive and strict public health measures in the face of an outbreak, in which case, the immunity of the human body plays a significant role.

It is widely accepted that physical activity (PA) is an essential part of a healthy and high-quality life. Apart from the benefits associated with preventing overweight, systemic inflammation, and chronic non-communicable diseases, regular physical activity (RPA) helps to reduce the spread of communicable diseases, including viral diseases [5,6]. Moreover, PA is beneficial to metabolic health as well as immune health, which decreases the risk of infectious complications [7,8,9,10]. Therefore, regular physical activity seems to be an effective measure for the prevention of viral infections—especially COVID-19 [11,12]. However, there remains a lack of sufficient, global, and in-depth theoretical analysis and empirical evidence. Hence, could regular physical activity reduce the possibility of being infected with COVID-19 and help to fight against the pandemic?

Since the outbreak of COVID-19, in addition to research on the origins [13,14,15], pathology [16,17], characteristics [18,19,20], and pharmacological and non-pharmacological interventions [21,22,23] of COVID-19, a large number of studies have focused on the correlation between physical activity and the disease. Some scholars have discussed the negative impact of the COVID-19 pandemic on physical activity due to the implementation of preventive and control measures such as lockdowns, which made it easier to lead a sedentary life during the pandemic [6,16,24,25,26], adversely affecting people both psychologically [27] and physiologically [6,28]. Other studies conducted during the COVID-19 outbreak report that physical activity can boost individual immunity [29,30,31,32], alleviate the negative emotions such as anxiety and depression associated with public health interventions such as quarantine and isolation [33,34], and reduce COVID-19-associated morbidity and mortality rates [35,36]. Some articles from the reverse perspective confirm that lack of physical exercise during the pandemic leads to worse COVID-19 outcomes [37].

However, some issues in the existing literature still need to be better addressed. First, most of the existing studies are qualitative [29,31,32,34,36,38] or based on cross-sectional or longitudinal data [30,33,35,37,39], and there is a lack of rigorous, quantitative analyses using causal inference with panel data. Moreover, few studies have explored the influencing mechanisms of physical activity on COVID-19. Limited by difficulties such as the availability of data, there are few empirical studies on how physical exercise affects the course of COVID-19 infection [29]. Second, most of the existing literature discusses the influence of engaging in physical activity during the pandemic on COVID-19 outcomes [35,37]. However, quality of life during the pandemic was severely affected by the implementation of a series of public health measures aimed at maintaining social distance, and fitness venues suspended their activities to reduce congregation, causing physical activity to suffer negative impacts from the pandemic as well. This inevitably raises endogenous issues of reverse causality [40,41], leading to biased estimation results and a failure to accurately identify the effects of physical activity [42,43].

In addition to the two aforementioned issues, it has been revealed that the spread of COVID-19 is also closely related to factors including human behavior patterns [44,45], public health interventions [46,47], and socioeconomic conditions [44]. However, limited by the available data and statistical approaches, there are few existing studies that take these factors into account when performing statistical analysis. The omission of such important variables, called issues of omitted variables in causal inference, necessarily leads to biased estimates [41]. In addition, most existing studies select local areas as the study area [37,48], making it difficult to capture the regional heterogeneity of the effect.

This study aims to fill the research gaps mentioned above. In this article, we investigate the effects and influencing mechanisms of regular physical activity on the COVID-19 pandemic. Daily epidemic-related panel data of 279 prefecture-level cities in mainland China were collected from 1 January to 17 March 2020. A two-way fixed-effects model in ordinary least squares (OLS) regression [11,42,43,46,47,49,50] was used to identify the causal relationship between physical activity and COVID-19, while also considering factors such as patterns of human behavior and socioeconomic conditions. To avoid biased estimation results due to endogeneity, the number of sports establishments was taken as an instrumental variable (IV) for physical activity, and a two-stage least squares (2SLS) estimation was performed [42,43,50]. Unlike other studies, we focus more on the role of the improvement of individual immunity by regular physical activity before the outbreak in epidemic prevention.

## 2. Conceptual Framework

There is a consensus in the existing literature that the immune system plays a significant role in susceptibility to COVID-19, along with its progression and outcomes [29], since the immune system identifies the virus and produces cytokines to inhibit viral replication and protect uninfected cells [1,51,52,53,54,55]. To start, cells in the innate immune system identify pathogens through pattern-recognition receptors (PRRs), which can stimulate the inflammatory response and activate chemotactic immune cells to drive phagocytosis and the expression of inflammatory cytokines [56]. Then, antiviral cytokines affect the activities of macrophages and lymphocytes to protect uninfected cells from viral replication and activate the adaptive immune system [1,53]. Macrophages and dendritic cells activate the adaptive immune system, and T-helper (Th) lymphocytes—primarily Th1—help stimulate the adaptive response and release cytokines, attracting monocytes and neutrophils, and facilitating other cascades of pro-inflammatory molecules that magnify the immune response [1,56]. It is widely believed that the factors that are related to a worse prognosis of infection [54] include imbalanced innate and adaptive immune responses, mainly featuring symptoms such as cytokine storm and lymphopenia [55], as well as disorders in coagulation and host-related conditions [9]. Therefore, viral infections are influenced by the individual immune system, which is affected by factors including genes, physical condition, and age.

As one of the key components of a healthy lifestyle, the significant role of physical activity in disease prevention and control has been widely discussed in the literature on clinical medicine and public health. It is believed that physical exercise is not only related to non-communicable chronic diseases such as heart disease, stroke, hypertension, osteoporosis, non-insulin-dependent diabetes mellitus, and depression [57,58,59,60,61], but also contributes significantly to the prevention, control, and diagnosis of infectious diseases [62,63]. 

It has been indicated that physical activity can significantly alter the immune system and have a beneficial effect on the response to viral infections [5,64,65,66]. Regular physical activity (RPA) increases immune immunovigilance and enhances immune competence, which helps control pathogens [67]. This becomes even more important in elderly people due to their immunosenescence and susceptibility to severe infections [68]. When an individual regularly engages in physical activity, the activity of anti-inflammatory cytokines in their bodies is enhanced, while that of pro-inflammatory cytokines is reduced [67,69,70], so that in case of inflammation, the immune system of the body can respond in a timely manner to remove diseased cells and tissues from the body and reduce the risk of infection. In addition, physical activity leads to a healthier cardiovascular system and metabolism [71], as well as a more favorable response to vaccines [67], and reduces the risk, duration, and severity of viral infections [5].

Theoretically, the improvement of the immune system induced by regular physical activity helps to reduce the severity of COVID-19 infection, so regular physical activity is considered to be an important immunomodulatory and non-pharmacological intervention. However, it has been shown that the regulation of exercise-related immune responses is dependent on factors including the regularity, intensity, duration, and type of exercise [5]. Participating in regular, moderate-intensity physical exercise promotes cellular immunity [64], while strenuous activities can cause immunosuppression, increasing the risk of infections [69]. Excessive physical exercise—especially during a pandemic—may lead to serious disease as a result of changes in the immune system [67]. This is because during strenuous exercise, in order to reduce damage to muscle tissue, the body produces large amounts of Th2 anti-inflammatory cytokines, which can lead to immunosuppression and increase the opportunity for infection [69,72]. In addition, it is believed that people who remain active throughout their lives exhibit less pronounced immunosenescence characteristics, which is likely to help prevent the development of complications after being infected by the SARS-CoV-2 virus [73]. Therefore, on the basis of the theoretical analysis above, the following two hypotheses are put forward:

**Hypothesis** **1.**  *There is a negative correlation between regular physical activity and confirmed COVID-19 cases. The greater the number of people who regularly engage in physical activity in a city, the fewer COVID-19 cases there are*.

**Hypothesis** **2.**  *Engaging in regular physical activity decreases the possibility of being infected by strengthening individual immunity; that is, the improvement of the immune system is the mechanism by which physical activity affects the COVID-19 pandemic*.

## 3. Materials and Methods

### 3.1. Empirical Strategy

#### 3.1.1. Baseline: Ordinary Least Squares Regression

In order to test the theoretical hypotheses, we performed ordinary least squares regression [11,43,47,49] to explore the effect of physical activity on COVID-19. To avoid biased estimators led by omitted variables, a two-way fixed-effects model, which is widely applied in the estimation of panel data [43,46], was used to control the time-invariant individual heterogeneity and the individual-invariant time heterogeneity. The empirical model is as follows:

(1) $$case_{i,t}=\alpha +\beta_{1}RPA_{i}+\gamma X_{it}+\delta_{c}+\delta_{t}+\varepsilon_{i,t}$$

where $Cases_{i,t}$ is the dependent variable, which represents the cumulative number of confirmed COVID-19 cases in city i on date t.

Considering that the average incubation period of COVID-19 is 5.2 days [74], we used the 5th lead of reported cases as a proxy variable for actual cases, as follows:

(2) $$Reported\;Cases_{i,t}=Actual\;Cases_{i,t+5}$$

where $RPA_{i}\;$ is the independent variable, indicating the number of people who regularly participated in physical activity in city *i* in 2019—that is, the sporting population:

(3) $$RPA_{i}=total\;population_{i}\times proportion\;of\;the\;sporting\;population_{i}$$

where $total\;population_{i}$ denotes the total population in city *i* in 2019, while $proportion\;of\;the\;sporting\;population_{i}$ denotes the proportion of the population who regularly participated in physical exercise in city *i* in 2019. The population regularly participating in physical exercise is defined as those who participate in moderate- or higher-intensity physical exercise three or more times per week, with each session lasting more than 30 min.

In 2014, China raised “national fitness” as a national strategy for the first time; then, in 2015, China put forward the “Healthy China” strategy for the first time, and issued a series of policies and action plans to promote the deep integration of national fitness and national health, including the National Fitness Program and the National Fitness Program Outline. In response to the national policy, the provinces issued action plans to implement and promote the national fitness strategy, and counted the sporting population of each prefecture-level city to better grasp and evaluate the effect of the implementation of the National Fitness Program. The proportional sporting population used in this paper is derived from the official statistics of the National Fitness Report, the National Fitness Development Survey Bulletin, and the National Fitness Action Plan issued by each province.

$X_{i\;}$ represents a set of control variables, including public health measures (measure), information dissemination (information), effective distance to Wuhan (effective distance), density of population (pop density), and passenger traffic (traffic). $\delta_{c}$ represents the provincial fixed effect, which is used to control the provincial characteristics that do not change with time; $\delta_{t}$ represents the temporal fixed effect, which is used to control the time factors that do not change with the individual; $\varepsilon_{i,t}$ is the error term; and the standard deviation is estimated using cluster-robust standard error [75].

It is emphasized that the linear model is the most classical modeling paradigm of the econometrics model. We linearized the nonlinear relationship by taking a logarithm to facilitate parameter estimation [46,47]. In addition, we also constructed the polynomial regression model in robustness and estimated the coefficients of quadratic and cubic terms of RPA.

#### 3.1.2. IV Approach: Two-Stage Least Squares Regression

To address the inevitable endogeneity issue caused by reverse causality and omitted variables, the baseline results were re-estimated using a two-stage least squares (2SLS) regression adopting an instrumental variable approach [41]. A valid instrumental variable should meet two conditions: first, the instrumental variable directly affects the number of regular physical activity participants (relevance condition); second, the instrumental variable is not correlated with the error term—that is, the instrumental variable only affects the cumulative number of COVID-19 cases through the regular physical activity participants (exogeneity condition). Based on existing studies, we selected access to exercise opportunities as an instrumental variable [42,43,50]. Specifically, we selected the number of fitness and sports establishments to characterize exercise opportunities as the instrumental variable (IV) for the independent variable (physical activity). It is believed that a sufficient number of sport and fitness establishments implies easier access to or greater emphasis on participation in physical activities, helping to meet the growing demand for sports, and the number of people involved in PA can be increased, satisfying the requirement of relevance, as also verified in Section 4.1. Furthermore, the number of sports establishments is not directly correlated with the number of COVID-19 cases, indicating that it also meets the requirement of exogeneity, making it a reasonable choice as an instrumental variable for physical activity.

#### 3.1.3. Mechanism Exploration: Mediating Effect Model

In this conceptual framework, it is proposed that engaging in regular physical activity improves individual immunity, thereby reducing the number of COVID-19 cases. Therefore, improved immunity is the mediating variable of this framework. To further examine the mechanism of influence, the mediating effect model [76] was performed as follows.

First, we examined whether the increase in the number of people participating in regular physical activity reduces the number of confirmed COVID-19 cases:

(4) $$case_{it}=\alpha +\beta_{1}RPA_{i}+\gamma X_{i,t}+\theta t+\delta_{c}+\delta_{t}+\varepsilon_{i,t}\;$$

Second, we investigated whether regular physical activity improves the immunity of residents:

(5) $$immunity_{i}=\alpha +\beta_{1}RPA_{i}+\gamma X_{i,t}+\theta t+\delta_{c}+\delta_{t}+\varepsilon_{i,t}$$

Third, both the sporting populations and individual immunity were introduced into the model:

(6) $$case_{it}=\alpha +\beta_{1}RPA_{i}+\beta_{2}immunity_{c}+\gamma X_{i,t}+\theta t+\delta_{c}+\delta_{t}+\varepsilon_{i,t}$$

where $immunity_{i}$ represents the total instances of diagnosis and treatment in the hospital of city i in 2019, as a proxy variable for individual immunity. Due to the limitations of the available data, it is assumed that a city with relatively strong individual immunity has fewer instances of diagnosis and treatment in the hospital.

### 3.2. Data Collection

In this study, the dataset was collected from 279 prefecture-level cities in mainland China, and the time span of the sample was from 1 January to 17 March 2020. The reported confirmed COVID-19 cases were taken from the national and provincial health commissions. The proportional sporting population was collected from the National Fitness Action Plan, National Fitness Development Survey Bulletin, and National Fitness Report released by each province and city. The data on total instances of diagnosis and treatment in the hospital were obtained from the *China Health Statistical Yearbook*. The original data on public health measures were sourced from the relevant announcements of the emergency headquarters for the prevention and control of COVID-19 in each city. Using big data-mining technology, the data on population migration were sourced from the Baidu Migration website, and the data on search term attention were sourced from the Baidu Index website. The data on population density and passenger traffic were sourced from the *China Urban Statistical Yearbook*. The descriptive statistics of the variables are presented in Table 1.

## 4. Results

### 4.1. Does Regular Physical Activity Matter?

Table 2 presents the baseline regression results. The dependent variable is the number of confirmed COVID-19 cases, and the independent variable is the number of people engaged in regular physical activity (RPA). Column 1 reports the results of the ordinary least squares (OLS) estimation with a two-way fixed-effects model, and Columns 2–4 report the results of the two-stage least squares (2SLS) estimation using the number of fitness and sports establishments as an instrumental variable. It can be seen that the coefficient of RPA is significantly negative, indicating that the increase in engagement in regular physical activity leads to a significant decrease in the number of confirmed COVID-19 cases—that is, regular physical exercise can effectively reduce the probability of being infected by COVID-19, indicating that Hypothesis 1 is confirmed.

### 4.2. Robustness

In this study, the following four methods were used to perform robustness tests. First, since Hubei Province was the first province in China to suffer from the COVID-19 epidemic, with the largest number of confirmed cases, we re-estimated the coefficients after excluding the sample of Hubei Province, and the results are reported in Column 1 in Table 3. Second, considering that there were few cases in most cities in January, we re-estimated the coefficients after excluding the sample from January, and the results are reported in Column 2 of Table 3. Third, in the baseline regression, the incubation period was assumed to be 5 days. In terms of robustness, the results were re-estimated under the assumption that the incubation period is 4 days and 6 days, and the results are presented in Columns 3 and 4, respectively. Fourth, we added the polynomial of RPA into the baseline model to explore the possible non-linear relationship, and the result is reported in Column 5. It can be seen in Columns 2–4 that the coefficient of RPA is still negative and statistically significant, proving the robustness of the results, and neither the quadratic term nor cubic term of RPA is statistically significant.

### 4.3. Regular Physical Activity, Immunity, and COVID-19

Furthermore, the influencing mechanism of RPA on the COVID-19 pandemic was examined using Equations (4)–(6). In Table 4, Column 1 presents the estimated results of Equation (4), Column 2 shows the results of Equation (5), and Column 3 reports the results of Equation (6). Column 2 indicates that regular physical exercise improves the individual immune system, and Column 3 introduces the mediating variable (immunity) based on Column 1. It is revealed that the coefficient of the independent variable decreases after introducing the mediating variable, indicating that the improvement of individual immunity is the influencing mechanism by which regular physical activity affects the number of COVID-19 cases. Regular physical activity significantly improves individual immunity, which, in turn, leads to a reduction in the probability of being infected with COVID-19, suggesting that Hypothesis 2 is confirmed.

### 4.4. When and Where Is Physical Activity More Important?

After the positive role of regular physical activity in the prevention of COVID-19 is confirmed, the question of under what conditions the role of regular physical activity becomes more important remains to be clarified. To address this issue, we divided the whole sample into two groups of high and low according to medical resources, residents’ awareness of protection, and the intensity of implementation of public health measures, and performed subsample regression to explore the heterogeneity of the effects. Columns 1 and 2 in Table 5 report the estimated results of subsamples according to medical resources, Columns 3 and 4 report the results according to residents’ awareness of protection, and Columns 5 and 6 report the results according to the intensity of implementation of public health measures. It can be seen that the effect of regular physical activity is greater when medical resources are relatively scarce, residents’ awareness of prevention is relatively weak, and public health measures are not fully implemented. The results of this exploration of heterogeneity further demonstrate the positive significance of regular physical activity, suggesting that not only should physical activity be actively practiced during the pandemic, but also long-term regular exercise habits should be consciously cultivated to improve the ability of the individual immune system to better cope with sudden outbreaks of emerging infectious diseases.

## 5. Discussion

This study investigated the influence and mechanisms of regular physical activity on the COVID-19 pandemic. The results indicate that the greater the number of people who engage in regular physical activity in the city, the fewer confirmed cases of COVID-19 there are. In addition, engaging in regular physical activity improves individual immunity, which, in turn, leads to a reduction in the probability of infection. Our findings are consistent with those of other studies, indicating that physical activity plays a significant role in the spread of COVID-19 [6,29,30,31,32,33,34,35,36,37,38,39]. Compared to these studies, we focused more on the significance of long-term, regular physical activity before the pandemic. In addition, based on the existing literature, we further empirically explored the influencing mechanisms of regular physical activity on the pandemic, providing new empirical evidence for the health benefits of physical activity.

It can be seen that the majority of the existing studies discussing the influence of physical activity on the outbreak are qualitative [29,31,32,34,36,38] or utilize cross-sectional or longitudinal data [30,33,35,37,39], but they rarely consider and address potential endogeneity. To make the statistical analysis more rigorous, we used daily city-level statistical panel data from China, used the two-way fixed-effects model of multiple linear regression, and selected the number of fitness and sports establishments as the instrumental variable of the independent variable to avoid biased estimates caused by endogeneity.

The transmission mechanism of COVID-19 is complicated, and considering only a single factor cannot accurately identify the causal relationship. Therefore, when analyzing the effects of regular physical activity on COVID-19 infection, as many potential influencing factors as possible should be controlled. In this study, we took into account factors including public health measures, patterns of human behavior, and socioeconomic conditions. It is believed that effective distance is a more decisive factor compared with geographical distance [45], so we measured the indicator of effective distance and introduced it into the empirical model. We found that the closer the effective distance to Wuhan—which was the first city in China in which COVID-19 was reported—the greater the number of COVID-19 cases. The adoption of rapid and strict public health interventions can significantly contain the further development of the pandemic and mitigate the spread. Based on the method proposed by Lin et al. [47], we further collected and evaluated the public health measures adopted in China. The results show that the coefficient of public health measures is significantly negative, implying that the implementation of public health interventions is a key factor in curbing the outbreak. The areas with better public health intervention tend to have fewer confirmed cases of COVID-19.

There are studies investigating the influence of physical activity on the COVID-19 outbreak in China [33,48]; most of them use local areas as the research sample, or cross-sectional data, such as data from the questionnaire. Since there are limitations in the sample selection and data structure, it is difficult to further explore the heterogeneity of effects—that is, to answer the question of when and where physical activity is more important. Therefore, a heterogeneity analysis was performed to further explore the effects of regular physical activity under different levels of medical resources, residents’ awareness of protection, and the intensity of public health measures, so as to address this issue. It was revealed that the negative impact of physical activity on the pandemic is more pronounced in the absence of adequate medical resources, strong awareness of prevention among residents, and fully implemented public health measures.

Such a state of affairs occurs more often in the initial stages of the outbreak of emerging infectious diseases. In the face of a sudden outbreak, sufficient medical resources are often not available. Due to the limited understanding of emerging infectious diseases, it is likely that residents did not have sufficient awareness of protection in the early stages of the epidemic. At the same time, it is difficult for the government to quickly implement public health measures. At this stage, the probability of residents being infected depends more on their physical condition—especially individual immunity. The importance of regular physical activity is particularly prominent at this time, further emphasizing the importance of participating in physical activity. In conclusion, participating in regular physical activity for a long period of time can help strengthen our immune system and protect us from infection—especially in the absence of timely and effective drug and non-drug interventions in the initial stages of a pandemic. Moreover, it has been shown that excessive exercise during an epidemic may adversely affect the immune system [67,69]. Therefore, it seems that developing good exercise habits consisting of regular physical activity at ordinary times is more effective than starting it after the outbreak of a pandemic.

There are still some limitations to this study that need to be further explored. First, in this paper, we used the total instances of diagnosis and treatment in the hospital as a proxy variable for individual immunity, which was the first attempt to characterize immunity using prefecture-level data. In future research, we will strive to explore the selection of more appropriate proxy variables to capture the immunity and physical fitness of the residents. In addition, it appears that personal characteristics, including age and gender, also have an influence on susceptibility to COVID-19 [27,77], as well as the effects of physical activity on the immune system [67], so more detailed microdata are needed to further explore this issue. In future research, it will be necessary to combine statistical data and questionnaire data in order to draw more reliable conclusions. At the same time, more in-depth interdisciplinary cooperation with experts in other fields is needed to further study the micro-mechanisms of the influence of physical exercise on COVID-19 from the perspective of fields such as sports science, exercise immunology, and public health, and draw clearer and more effective conclusions.

## 6. Conclusions

Our research provides empirical evidence for the influence and mechanism of regular physical activity on the COVID-19 pandemic. First, a two-way fixed-effects model in ordinary least squares (OLS) regression was applied to explore the effects of regular physical activity on the number of COVID-19 cases. To avoid biased estimation results due to endogeneity, the number of fitness and sports establishments was taken as an instrumental variable (IV) for physical activity, and two-stage least squares (2SLS) estimation was performed. Then, the mediating effect model was applied to examine the mechanisms of the influence of physical activity on the pandemic, and it was revealed that regular physical activity significantly improves individual immunity, which, in turn, reduces the probability of being infected with COVID-19. Furthermore, we explored the heterogeneity of the influence, and found that the negative impact of physical activity on the pandemic is more pronounced in the absence of adequate medical resources, strong awareness of prevention among residents, and fully implemented public health measures. The exploration of heterogeneity further demonstrates the positive significance of regular physical activity, suggesting that not only should physical activity be actively practiced during a pandemic, but also long-term regular exercise habits should be consciously cultivated in order to improve the ability of the individual immune system to better cope with sudden outbreaks of emerging infectious diseases.

## Figures and Tables

**Table 1:** Descriptive Statistics.

Type	Name	Obs	Mean	Std. Dev.	Min	Max
dependent variable	case	19,989	2.1221	1.8792	0	10.8199
independent variable	RPA	19,989	3.4891	0.8286	1.5586	6.8084
control variable	measure	19,989	5.2923	3.9634	0	10
	pop density	19,989	4.7855	0.7808	2.8332	7.8047
	information	19,989	0.3730	0.4836	0	1
	traffic	19,989	6.7557	1.0995	4.1431	10.4860
	effective distance	19,989	5.7160	1.8739	0	7.7846
instrumental variable	sports establishments	19,989	2.7952	1.9651	0	6.4061
mediating variable	immunity	19,989	18.2574	0.6818	16.0217	19.5768

**Table 2:** Baseline result.

	(1)	(2)	(3)	(4)
	OLS	2SLS	First Stage	Reduced Form
	Case	Case	RPA	Case
RPA	−0.4376 ***	−3.0335 ***		
	(0.0352)	(0.2918)		
IV: sports establishments			0.0004 ***	−0.0011 ***
			(0.0000)	(0.0001)
public health	−0.1699 ***	−0.5043 ***	−0.0993 ***	−0.0948 ***
	(0.0250)	(0.0409)	(0.0057)	(0.0287)
pop density	0.5948 ***	3.2329 ***	1.0382 ***	0.0983 ***
	(0.0408)	(0.3030)	(0.0044)	(0.0220)
information	−0.2441 ***	0.1090 ***	0.0835 ***	−0.2015 ***
	(0.0209)	(0.0362)	(0.0048)	(0.0238)
traffic	0.0940 ***	0.1397 ***	−0.0167 ***	0.1879 ***
	(0.0131)	(0.0177)	(0.0035)	(0.0174)
effective distance	−0.3847 ***	−0.2727 ***	0.0329 ***	−0.3778 ***
	(0.0061)	(0.0118)	(0.0014)	(0.0070)
Control Variables	YES	YES	YES	YES
Province FE	YES	YES	YES	YES
Time FE	YES	YES	YES	YES
Observations	19,989	19,989	19,989	19,989
R-squared	0.697	0.572	0.725	0.695
F-statistics			292.668	

Standard errors in parentheses, *** *p* < 0.01, ** *p* < 0.05, * *p* < 0.1.

**Table 3:** Robustness.

	(1)	(2)	(3)	(4)	(5)
	Exclude Hubei Province	Exclude January	Incubation = 4	Incubation = 6	Add Polynomial
RPA	−0.2082 ***	−0.7163 ***	−0.4361 ***	−0.4390 ***	−0.5535 ***
	(0.0312)	(0.0345)	(0.0343)	(0.0362)	(0.0275)
quadratic term					−0.0003
					(0.0001)
cubic term					−2.5364
					(1.1169)
Control Variables	YES	YES	YES	YES	YES
Province FE	YES	YES	YES	YES	YES
Time FE	YES	YES	YES	YES	YES
Observations	19,125	11,372	19,989	19,989	19,989
R-squared	0.734	0.733	0.703	0.690	0.792

Standard errors in parentheses, *** *p* < 0.01, ** *p* < 0.05, * *p* < 0.1.

**Table 4:** Mechanism test.

	(1)	(2)	(3)
	Case	Immunity	Case
RPA	−0.4376 ***	−0.0008 ***	−0.5032 ***
	(0.0352)	(0.0001)	(0.0357)
immunity			0.0655 ***
			(0.0081)
Control Variables	YES	YES	YES
Province FE	YES	YES	YES
Time FE	YES	YES	YES
Observations	19,989	19,989	19,989
R-squared	0.697	0.475	0.698

Standard errors in parentheses, *** *p* < 0.01, ** *p* < 0.05, * *p* < 0.1.

**Table 5:** Heterogeneity exploration.

	(1)	(2)	(3)	(4)	(5)	(6)
	Medical Resource_H	Medical Resource_L	Prevention Awareness_H	Prevention Awareness_L	Public Health_H	Public Health_L
RPA	−0.4052 ***	−0.7668 ***	−0.2514 ***	−0.7213 ***	−0.4285 ***	−0.8546 ***
	(0.0411)	(0.0534)	(0.0366)	(0.0354)	(0.0453)	(0.0472)
Control Variables	YES	YES	YES	YES	YES	YES
Province FE	YES	YES	YES	YES	YES	YES
Time FE	YES	YES	YES	YES	YES	YES
Observations	10,080	9909	9259	10,730	10,008	9981
R-squared	0.718	0.692	0.610	0.621	0.695	0.713

Standard errors in parentheses, *** *p* < 0.01, ** *p* < 0.05, * *p* < 0.1.

## Data Availability

Data available on request due to restrictions, e.g., privacy or ethical.
